# Changes in adolescents' intake of sugar-sweetened beverages and sedentary behaviour: Results at 8 month mid-way assessment of the HEIA study - a comprehensive, multi-component school-based randomized trial

**DOI:** 10.1186/1479-5868-8-63

**Published:** 2011-06-17

**Authors:** Mona Bjelland, Ingunn H Bergh, May Grydeland, Knut-Inge Klepp, Lene F Andersen, Sigmund A Anderssen, Yngvar Ommundsen, Nanna Lien

**Affiliations:** 1 Department of Nutrition, Faculty of Medicine, University of Oslo, Oslo, Norway; 2 Department of Sports Medicine, Norwegian School of Sport Sciences, Oslo, Norway; 3 Department of Coaching and Psychology, Norwegian School of Sport Sciences, Oslo, Norway

## Abstract

**Background:**

Inconsistent effects of school-based obesity prevention interventions may be related to how different subgroups receive them. The aim of this study was to evaluate the effect of an intervention program, including fact sheets to parents and classroom components, on intake of sugar-sweetened beverages (SSB) and screen time. Further, to explore whether potential effects and parental involvement varied by adolescents' gender, weight status (WS) and parental educational level.

**Methods:**

In total, 1465 11-year-olds participated at the pre-test and the 8 month mid-way assessment of the HEIA study. Parents (n = 349) contributed with process evaluation data. Self-reported intake of SSB was collected from the 11-year-olds assessing frequency and amount, while time used on watching TV/DVD and computer/game-use (weekday and weekend day) were assed by frequency measures. Data on awareness of the intervention and dose received were collected from parents. Covariance analyses (ANCOVA) were conducted testing for effects by gender and for moderation by WS and parental education.

**Results:**

Time spent on TV/DVD (week p = 0.001, weekend p = 0.03) and computer/game-use (week p = 0.004, weekend p <.001), and the intake of SSB during weekend days (p = 0.04), were significantly lower among girls in the intervention group compared to the control group girls after 8 months. Girls' WS did not moderate these findings. However, no significant effects of the intervention were found for boys, but moderation effects were found for WS (week days: TV/DVD, p = 0.03 and computer/games, p = 0.02). There were no moderating effects of parental education for neither boys nor girls with respect to intake of SSB, time used for watching TV/DVD and computer/game-use. Parental awareness of the intervention was significantly higher among the parents of girls, while the parents of boys were more satisfied with the fact sheets.

**Conclusions:**

The preventive initiatives appeared to change behaviour in girls only. This study suggests that exploration of potential beneficial or negative effects of intervention in subgroups is important. In formative evaluation of obesity prevention studies it seems warranted to include issues related to gender, WS and parental involvement in order to enhance the effectiveness of preventive initiatives.

## Background

Interventions to prevent unhealthy weight gain should aim at making a change in energy balance related behaviours (EBRB) [1]. The consumption of sugar-sweetened beverages (SSB), television viewing and computer use are behaviours that have been associated with increased risk for obesity [2]. Lack of effective school-based obesity prevention interventions [3,4] has initiated a debate about the best intervention strategies and evaluation designs [4,5]. Intervention strategies tailored to specific subgroups (like gender) [3,6], including family components [7,8], and evaluated by the target groups [9] seem needed in order to examine for whom and why obesity prevention programmes works.

Schools are often used as a setting for implementing interventions developed to reduce the prevalence of obesity in children and adolescents, because it offers continued and intensive contact with a large population across ethnic and socio-economic groups [3,7]. However, including the home- and family environment could increase the effectiveness of school-based prevention of obesity [10,11], and such interventions have been requested [7,8]. Process evaluation of environment-focused interventions is also requested, including the social environment [4]. Both dietary habits and sedentary behaviours are mainly performed in the home and family environment [12,13], with parents being key persons in children and adolescents' social environment. Nevertheless, the effects of parental involvement in obesity prevention programs are still unclear [8].

Obesity risk may differ across subgroups, and intervention strategies may not be equally effective across these groups [14]. Gender is the most convincing and most frequently examined moderator of school-based interventions aimed at EBRB, and the interventions seem to work better for girls than for boys [15,16]. It may be that in early adolescence, boys and girls respond differently to various intervention strategies [3]. Weight status (WS) and socio-economic status (SES) have not been shown to be consistently moderators of EBRB [15,16]. The WS of children and adolescents may affect their dietary habits and sedentary behaviours of which TV-viewing is an example [12]. Watching TV may be a risk factor for obesity, but the causal arrow may be backward; that obesity itself increases TV-viewing [17,18]. Lower SES children have a higher risk of obesity, and parental education has been found to be inversely associated with sedentary behaviours and consumption of SSB in adolescents [19,20]. More research is needed exploring further the moderating effect of WS and SES in obesity prevention studies [15,16].

Process evaluation data might serve to better interpret the intervention effects, but a limited number of published intervention studies report on process evaluation, including data on implementation quality and quantity of exposure [8,9]. In health promoting interventions parental involvement could be assessed by the awareness of the intervention components, the dose received and the satisfaction with the components [21-23]. Conducting process evaluation is important in order to identify the reach and dose received by the participants [24,25], and indices of dose received can be assessed in terms of both intervention exposure and satisfaction [26].

The overall goal of the HEalth In Adolescents (HEIA) study was to design, implement and evaluate a comprehensive, intervention program to promote healthy weight development among young adolescent school-children (11-13 year olds). The targeted changes in the behaviours were to decrease consumption of SSB and sedentary behaviour, and to increase the physical activity and the consumption of fruit and vegetables [27]. In this study, the two behaviours to be reduced were explored in relation to the important issues raised in the literature and summarised above.

The aim of this paper was three-fold. Firstly, to determine if a multi-component health promotion intervention targeting 11-12 year olds influenced their consumption of SSB, television viewing and/or computer/game-use. Secondly, the aim was to explore whether the results varied by gender, adolescent WS or by parental educational level. Finally, the aim was to assess whether parental involvement differed by parental educational level or by the adolescents' gender or WS.

## Methods

### Study design and subjects

**Figure 1:**
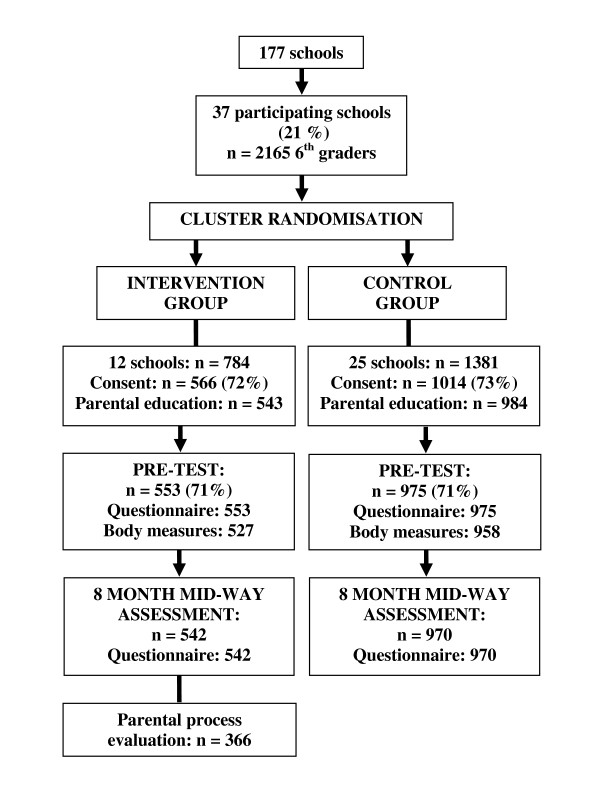
**Flow diagram of recruitment, randomization and participation of adolescents and parents in the HEIA study**.

Eligible schools were located in the Eastern part of Norway and had more than 40 pupils in 6^th ^grade. Such schools are mainly located in larger towns/municipalities, and 37 schools were recruited from the largest towns/municipalities in seven counties surrounding Oslo (Figure 1) [27]. All 6^th ^graders in these 37 schools (n = 2165) and their parents/legal guardians were invited to participate. Of these, 1580 returned a parent signed informed consent form for the adolescent. A cluster randomized controlled pre-post study design was used to evaluate the effectiveness of the intervention; 12 schools were randomly assigned by simple drawing to the intervention group and 25 to the control group. The pre-test data collection took place during four weeks in September 2007, while the 8 month mid-way assessment took place in May 2008.

The adolescents who participated in both data collections were included in this paper, as were the parents in the intervention schools who answered the process evaluation questions at the mid-way assessment (one questionnaire per pupil). A total of 1465 adolescents (92.7% of those 1580 returning consent) and 349 parents (82.5% mothers and 17.5% fathers, in total 65.7% of the parents in the intervention group) were included in the analyses. There were no significant differences in demographic and behavioural variables between those participating both at the pre-test and at the mid-way assessment compared to those lost to the mid-way assessment (n = 63).

The intervention program in the HEIA study consisted of a mixture of individual-, group-, and environmental level strategies and activities. Strategies and activities in the 6^th ^grade were: Lessons with student booklet, posters, weekly fruit and vegetable breaks and activity breaks in classroom, sports equipment for recess activities, active commuting, fact sheets for parents and an inspirational course for physical education teachers [27].

Ethical approval and research clearance was obtained from the Regional Committees for Medical Research Ethics and the Norwegian Social Science Data Service.

### Questionnaire data

The Internet based child questionnaire comprised mostly questions with pre-coded answer categories and could be completed in about 45 minutes. The parental process evaluation questionnaire (paper-pencil format) were sent home with the adolescent at the mid-way assessment, completed by one of the parents, returned to the teachers in a sealed envelope and collected from the schools by project staff. Process evaluation questions that tapped into the parents' perceived exposure and satisfaction are included in this paper.

### Behavioural outcomes

The intake of beverages was assessed by frequency (six categories, from never/seldom to every weekday) and amount (in glasses, four categories: from 1 glass to 4 glasses or more) for weekdays and by amount for weekends (in glasses, eight categories: from never/seldom to 7 glasses or more). Soft drinks with sugar and sugar-sweetened fruit drinks were the targeted beverages (summed and presented as SSB). Two questions assessed the number of hours spent on watching TV and/or DVD during a regular weekday and weekend day (six categories, 0.5 - 5 hours). Similarly, two questions assessed the number of hours spent on the computer, playing TV-games or other electronic games on a regular weekday and a weekend day, respectively (six categories, 0 to 4 hours). The test-retest correlation coefficients for the outcome measures were moderate to high (r = 0.46-0.78) [27].

### Process evaluation

Process evaluation is used to explore what happened in the intervention program, to what extent the intervention reached intended participants, and how that could affect program impacts or outcomes. Some of the elements in a process evaluation are reach and dose received [26]. Reach can be defined as participation rate and a quantification of how many within the intended target audience who participated in the intervention [26]. Dose received in the meaning of exposure are used to describe and quantify how much of the intervention that was received, whereas dose received related to satisfaction is used to describe and rate the participants' liking [26].

The process evaluation questions for parents comprised parental awareness of the intervention components/program activities for the adolescents at school (Have you heard of these components? Seven components, yes = 1/no = 0) as an indication of to which extend the adolescents talked about the project at home. The answers were summed and recoded into tertiles; low to moderate awareness (0-3 components), moderate to high (4-5) and high (6-7). Dose received was assessed with regards to the fact sheets handed out at school and sent home with the adolescent (Have you received or read the following fact sheets? Seven sheets and topics, three categories for each sheet: received = 1/read = 2/not aware of it = 0). The answers were summed and recoded into tertiles; low to moderate dose (0-7), moderate to high (8-13) and high (14). Parents also reported what they thought about the intervention (Overall, what do you think about the HEIA project in grade 6? Four response categories: did not like it at all = 0 to liked it very much = 3, those answering "I do not know the HEIA project" were excluded). The answers were combined into tertiles; low to moderate liking (0-1), moderate to high (2), and high (3). Finally, they were asked to give their opinion on the fact sheets (Overall, what do you think about the fact sheets? Three statements related to (1) appreciation of receiving the sheets, (2) interesting content and (3) useful tips, four response categories for each statement: not at all = 0 to a high degree = 3). The answers were summed and recoded into tertiles; low to moderate liking (0-6), moderate to high (7-8) and high (9).

### Weight status and parental education

The age- and gender specific body mass index (BMI) cut-off values proposed by the International Obesity Task Force [28] were used to categorize the adolescents as normal weight and overweight. Due to few obese adolescents (1.6%) these were included in the same group as the overweight adolescents in the analyses. Details of the anthropometrics of the participants and test-retest values of the measures have been reported elsewhere [27,29]. Parental education was collected as part of the informed consent form filled in by parents for the adolescent. Education was categorized into three levels: 12 years or less, between 13 and 16 years and more than 16 years. The parent with the longest education was used in the analyses, or else the one available.

### Data analysis

Clustering effects due to schools being the unit of recruitment was checked by the Linear Mixed Model procedure. Only 1-3% of the unexplained variance in the behaviours was on group level, and it was therefore decided to not conduct multilevel analysis.

The characteristics at the pre-test are presented as proportions (demographic variables), means and standard deviations (SD) (behavioural variables). Continuous variables were tested for differences between the intervention group and the control group with independent sample t-tests, and Chi-square test of proportions was used for categorical variables.

The effect of the intervention was determined using one-way ANCOVA with the mid-way value for the outcomes as the dependent variables, the experimental group as the independent variable and the pre-test values of the outcomes as covariates. The data were checked to ensure that there were no violations of the assumptions. Interaction effects by WS and parental educational level were tested in separate analyses as a second step, using two-way ANCOVA. To further explore gender differences by WS for the behavioural variables one-way ANCOVA were used. For secondary analyses a magnitude-based inference were made using a spreadsheet [30].

Chi-square test of proportions were used to assess whether the parental involvement in the intervention differed by gender, WS or parental educational level. The significance level was set at p <.05.

Data were analysed using SPSS Statistics, version 16 (IBM Corporation, New York, USA).

## Results

The pre-test characteristics of the control and intervention group are presented in Table 1 and Table 2. No significant differences were found between the groups with respect to demographic and behavioural variables.

**Table 1:** Pre-test characteristics (demographic) for the control and the intervention group in the HEIA study

		Pre-test		
		Control	Intervention	
		**n**^† ^**= 910**	**n**^† ^**= 510**	**p**

**Age **(mean (SD))		11.2 (0.27)	11.2 (0.26)	0.30
**Gender**				
Boys (%)		52.0	50.7	0.61
Girls (%)		48.0	49.3	
**Weight status**				
Normal weight (%)		84.9	88.5	0.06
Overweight (%)		15.1	11.5	
**Parental educational level**				
< 12 years (%)		32.0	26.3	0.07
13-16 years (%)		35.7	37.8	
> 16 years (%)		32.3	35.9	

p = Pearson Chi-Square/T-test (age)

^†^n vary slightly

**Table 2:** Pre-test characteristics (behaviours) for the control and the intervention group in the HEIA study

	Pre-test				
	Control		Intervention		
	**n**^† ^**= 881**		**n**^† ^**= 490**		

	**Mean**	**SD**	**Mean**	**SD**	**p**

**SSB, dl/d** **- week**	1.2	(1.7)	1.1	(1.6)	0.28
**- weekend**	2.3	(2.0)	2.3	(2.0)	0.76
**TV/DVD, hours/d** **- week**	1.5	(1.0)	1.5	(1.1)	0.65
**- weekend**	2.1	(1.2)	2.2	(1.3)	0.13
**Computer/games, hours/d** **- week**	1.1	(0.9)	1.1	(0.9)	0.16
**- weekend**	1.5	(1.1)	1.5	(1.1)	0.69

SSB = Sugar-sweetened beverages

p = T-test

^†^n vary slightly

The changes in outcome variables in the control and intervention groups from the pre-test to the mid-way assessment are summarized in Table 3. In the total sample, significant differences were found between the intervention group and control group in the number of hours watching TV/DVD during week days (p = 0.002) and weekend days (p = 0.04), and time spent on computer/games during weekend days (p = 0.003). Stratified by gender, the results showed effects for girls only. The girls in the intervention group spent significantly less time on watching TV/DVD and computer/game-use compared to the girls in the control group, and the intake of SSB during weekend days was significantly lower among the girls in the intervention group.

**Table 3:** Effects at 8 months mid-way assessment of the HEIA study, for all and by gender

	8 months mid-way assessment					8 months mid-way assessment									
	Total sample					Girls					Boys				
	Control		Intervention			Control		Intervention			Control		Intervention		
	Mean^†^	CI	Mean^†^	CI	p	Mean^†^	CI	Mean^†^	CI	p	Mean^†^	CI	Mean^†^	CI	p
	n^‡ ^= 840		n^‡ ^= 469			n^‡ ^= 416		n^‡ ^= 241			n^‡ ^= 424		n^‡ ^= 228		
**SSB, dl/d** **- week**	1.1	(1.0,1.2)	1.0	(0.9,1.1)	0.19	0.9	(0.8,1.0)	0.8	(0.6,0.9)	0.23	1.4	(1.2,1.5)	1.3	(1.1,1.5)	0.52
Group × WS					0.80					0.30					0.71
Group × PE					0.81					0.92					0.76
**- weekend**	2.4	(2.3,2.5)	2.3	(2.1,2.4)	0.20	2.1	(2.0,2.3)	1.9	(1.7,2.1)	**0.04**	2.6	(2.5,2.8)	2.6	(2.4,2.9)	1.00
Group × WS					0.77					0.83					0.97
Group × PE					0.35					0.47					0.77
**TV/DVD, hours/d** **- week**	1.6	(1.6,1.7)	1.5	(1.4,1.5)	**0.002**	1.6	(1.5,1.6)	1.3	(1.2,1.5)	**0.001**	1.7	(1.6,1.8)	1.6	(1.5,1.7)	0.20
Group × WS					**0.05**					0.36					**0.03**
Group × PE					0.26					0.83					0.26
**- weekend**	2.3	(2.3,2.4)	2.2	(2.1,2.3)	**0.04**	2.3	(2.2,2.3)	2.1	(1.9,2.2)	**0.03**	2.4	(2.3,2.5)	2.3	(2.2,2.5)	0.44
Group × WS					0.68					0.86					0.37
Group × PE					0.23					0.75					0.18
**Computer/games, hours/d** **- week**	1.2	(1.1,1.2)	1.1	(1.0,1.2)	0.06	1.0	(1.0,1.1)	0.9	(0.8,0.9)	**0.004**	1.3	(1.2,1.4)	1.3	(1.2,1.4)	0.76
Group × WS					**0.01**					0.08					**0.02**
Group × PE					0.31					0.34					0.73
**- weekend**	1.6	(1.6,1.7)	1.5	(1.4,1.6)	**0.003**	1.4	(1.3,1.5)	1.1	(1.0,1.3)	**<.001**	1.9	(1.8,1.9)	1.8	(1.7,1.9)	0.58
Group × WS					0.19					0.78					0.09
Group × PE					0.14					0.10					0.90

SSB = Sugar-sweetened beverages, Group = intervention and control, WS = weight status, PE = parental educational level

Analyses: Overall for all and by gender, one-way ANCOVA. Group × WS/group × PE: separate interaction analyses for weight status and for parental education, two-way ANCOVA

^†^Adjusted for pre-test behaviour

^‡^n vary slightly

Analyses of moderating effects by the adolescents' WS and parental education on pre-test to mid-way changes in the control and intervention groups, revealed an interaction of WS; number of hours spent on watching TV/DVD (borderline, p = 0.05) and computer/game-use during week days (p = 0.01) for the total sample. For boys, the same interactions were found (TV/DVD, p = 0.03 and computer/games, p = 0.02). No interactions were found for girls. Based on these findings we proceeded to explore gender differences by WS for the behavioural variables, and the stratified analyses are presented in Table 4. No moderating effect was found for parental education and no stratified analyses were conducted.

**Table 4:** Effects at 8 months mid-way assessment of the HEIA study, by gender and weight status

GIRLS	Normal weight		Overweight/ obese		BOYS	Normal weight		Overweight/ Obese	
	**Mean^†^**	**CI**	**Mean^†^**	**CI**		**Mean^†^**	**CI**	**Mean^†^**	**CI**

**SSB, dl/d** **- week**	n = 545		n = 92		**SSB, dl/d** **- week**	n = 550		n = 85	
Control	0.9	(0.8,1.0)	0.9	(0.6,1.3)	Control	1.4	(1.3,1.6)	1.1	(0.8,1.5)
Intervention	0.8	(0.7,1.0)	0.5	(0.0,1.0)	Intervention	1.3	(1.1,1.5)	1.2	(0.6,1.8)
**- weekend**	n = 572		n = 98		**- weekend**	n = 600		n = 90	
Control	2.1	(2.0,2.3)	2.0	(1.5,2.4)	Control	2.6	(2.5,2.8)	2.5	(2.0,3.0)
Intervention	1.9^#^	(1.7,2.1)	1.5	(0.9,2.2)	Intervention	2.6	(2.4,2.9)	2.5	(1.6,3.4)
**TV/DVD, hours/d** **- week**	n = 579		n = 100		**TV/DVD, hours/d** **- week**	n = 630		n = 93	
Control	1.6	(1.5,1.6)	1.6	(1.4,1.8)	Control	1.6	(1.5,1.7)	2.0	(1.7,2.2)
Intervention	1.3**	(1.2,1.4)	1.6	(1.3,1.9)	Intervention	1.5	(1.4,1.6)	2.4	(1.9,2.8)
**- weekend**	n = 570		n = 99		**- weekend**	n = 619		n = 93	
Control	2.2	(2.1,2.3)	2.3	(2.0,2.6)	Control	2.4	(2.3,2.5)	2.6	(2.4,2.9)
Intervention	2.1*	(1.9,2.2)	2.1	(1.7,2.5)	Intervention	2.3	(2.2,2.4)	2.9	(2.4,3.4)
**Computer/games, hours/d** **- week**	n = 578		n = 101		**Computer/games, hours/d** **- week**	n = 628		n = 94	
Control	1.0	(1.0,1.1)	1.0	(0.8,1.2)	Control	1.3	(1.2,1.4)	1.5	(1.2,1.7)
Intervention	0.8***	(0.7,0.9)	1.1	(0.8,1.3)	Intervention	1.2	(1.1,1.3)	2.0^#^	(1.5,2.4)
**- weekend**	n = 570		n = 100		**- weekend**	n = 624		n = 93	
Control	1.4	(1.3,1.5)	1.7	(1.4,1.9)	Control	1.8	(1.7,1.9)	2.0	(1.8,2.3)
Intervention	1.1***	(1.0,1.2)	1.4	(1.1,1.8)	Intervention	1.8	(1.6,1.9)	2.4	(1.9,2.9)

SSB = Sugar-sweetened beverages, Overweight including obese

One-way ANCOVA

^†^Adjusted for pre-test behaviours

*** p = <.001, ** p = 0.001, *p = 0.04, ^# ^p = 0.06

Among the normal weight girls there were significant differences between the intervention and control group for the sedentary behaviours. The normal weight girls in the intervention group spent significantly less time on watching TV/DVD and computer/game-use compared to the normal weight girls in the control group (Table 4). For intake of SSB during weekend days the results were borderline significant (p = 0.06). We found the same trends among the overweight/obese girls, except for use of computer/games during week days, but the differences between the intervention and control groups were not significant.

For the boys, no significant differences were found neither among the boys with normal weight nor the overweight/obese boys (Table 4). Even though not significant, the overweight/obese boys in the intervention group spent more time on watching TV/DVD and computer/game-use compared to the control group after the intervention. Time used for computer/games during week days was borderline significant among the overweight/obese boys in the intervention group compared to the overweight/obese boys in the control group (p = 0.06). We made a magnitude-based inference about the true effect of the intervention on computer/game-use in week days among overweight/obese boys, which provided the uncertainty in the effect as 95% confidence limits and as likelihoods that the true value of the effect represented a harmful, trivial or beneficial change in the experimental group compared with that in the control group. After log-transformation of the dependent variable adjusted for pre-test (daily hours used for computer/games on weekdays), the mean effect was expressed in standardized units (fraction of the between-subject standard deviation at pre-test). The smallest standardized change was assumed to be 0.20 [31]. There was an 85% chance that the true effect was positive, 15% chance that it was trivial, and 0.3% chance that it was negative (standardized difference in the mean as Cohen units = 0.43, confidence interval -0.01 to 0.87, p = 0.06). Thus the intervention likely produced an increase in time used for computer/game-use in week days in overweight/obese boys.

No significant differences were found in age, height, BMI, pubertal development, parental education and sedentary behaviours at pre-test between overweight/obese boys in the intervention group and overweight/obese boys in the control group. The only exception was time used for TV/DVD during weekend days which was higher among overweight/obese boys in the intervention group (p = 0.03) (data not shown).

No differences were found in parental involvement when stratifying by the adolescents' WS and the parental educational level. However, parental awareness of the intervention was significantly higher among the parents of girls, while the parents of boys were more satisfied with the fact sheets (Table 5).

**Table 5:** Parental involvement at 8 months mid-way assessment of the HEIA study

Parents		Daughter %	Son %	p		Normal weight %	Overweight/ obese %	p		12 years or less %	Between 13 and 16 year%	More than 16 years %	p
**Parental awareness**	n	169	145		n	263	33		n	70	119	117	
Low to moderate	104	23.7	44.1	**0.001**	98	33.1	33.3	0.86	100	22.9	36.1	35.0	0.33
Moderate to high	97	34.3	26.9		92	31.6	27.3		96	35.7	27.7	32.5	
High	113	42.0	29.0		106	35.4	39.4		110	41.4	36.1	32.5	
**Dose received**	n	164	139		n	253	32		n	64	118	113	
Low to moderate	88	25.6	33.1	0.36	83	28.9	31.2	0.67	85	37.5	25.4	27.4	0.10
Moderate to high	109	37.8	33.8		100	36.0	28.1		106	26.6	44.1	32.7	
High	106	36.6	33.1		102	35.2	40.6		104	35.9	30.5	39.8	
**Satisfaction** **- overall**	n	183	158		n	286	37		n	78	130	123	
Low to moderate	22	7.1	5.7	0.55	20	6.3	5.4	0.98	22	7.7	3.8	8.9	0.57
Moderate to high	174	53.0	48.7		166	51.4	51.4		169	51.3	51.5	50.4	
High	145	39.9	45.6		137	42.3	43.2		140	41.0	44.6	40.7	
**Satisfaction** **- fact sheets**	n	179	150		n	274	36		n	74	125	121	
Low to moderate	130	44.1	34.0	**0.01**	123	39.8	38.9	0.70	126	37.8	32.8	47.1	0.20
Moderate to high	81	18.4	32.0		78	24.5	30.6		80	24.3	26.4	24.0	
High	118	37.4	34.0		109	35.8	30.6		114	37.8	40.8	28.9	

Only adolescents and parents at the intervention schools were included in the analyses

p = Pearson Chi-Square

Parental awareness: parental awareness of program activities for adolescents at school

Dose received: fact sheets received by parents

Satisfaction - overall: parental satisfaction of the project in general

Satisfaction - fact sheets: parental satisfaction of the fact sheets for parents (appreciation of receiving them/interesting content/useful tips)

## Discussion

Data from the 8 month mid-way assessment indicated that girls in the intervention group spent significantly less time on watching TV/DVD and using computer/games compared to the girls in the control group, and the intake of SSB during weekend days was significantly lower among the girls in the intervention group. Girls' WS did not moderate these findings. No significant differences between the intervention and control group were found for outcome variables among the boys with normal weight or the overweight/obese boys, but moderation effects were found for WS (TV/DVD and computer/games during week days). There were no moderating effects of parental education for neither boys nor girls with respect to any of the three behaviours. The process evaluation showed that parental awareness was significantly higher among the parents of girls, while the parents of boys were more satisfied with the fact sheets. No other differences in the parental process evaluation were found.

The effects found were both in a desired direction (girls) and an undesired direction (overweight/obese boys). However, it may be questioned whether the effects were large enough to have any public health impact. One review suggests that in children an imbalance over time of about 2% (125 KJ or 15 minutes of play replaced by TV-viewing) may lead to obesity [32]. Based on these estimates two groups did benefit from the HEIA study. The decrease in intake of SSB among the overweight girls was 0.4 dl for week days and 0.5 dl for weekend days. This represents a decrease in calorie intake equal to 68-85 KJ per day (0.4 or 0.5 dl and 170 KJ/dl). By reducing the time used for TV/DVD during week days by 0.3 hours (about 18 minutes) and time used for computer/games during week days by 0.2 hours (about 12 minutes) among normal weight girls, the total sedentary screen time was reduced by 30 minutes, indicating a decrease in sedentary behaviour with a possible public health impact.

Further, we found that the overweight/obese boys in the intervention group had a non-significant tendency towards an undesired effect with regards to more time used for computer/games during week days compared to the overweight/obese boys in the control group (p = 0.06). By the use of magnitude-based inference as an alternative approach for this variable, we explored to what extent this change was of relevance. A confidence interval or p-value does not address the question of the clinical or practical importance of an outcome; a magnitude-based inference does [33]. It was possible to estimate the chances or probabilities that the true effect was harmful, trivial or beneficial, and the chances were estimated using the same assumptions about the outcome statistic as when estimating p-values or confidence intervals. The result indicated that the intervention likely produced an increase in time used for computer/game-use in week days in overweight/obese boys. This was an effect of clinical/practical importance, however, it was an unintended and undesired consequence of the intervention.

We can only speculate in why the overweight/obese boys did not respond to the intervention in a desired direction and why the overweight/obese girls did so. The same goes for why we found an overall effect in girls and not in boys. With respect to the former, it could well be that boys being overweight/obese show evidence of reactance by responding with less functional strategies (becoming more sedentary) when confronted with messages concerning healthy eating and enhanced physical activity [34]. As to the latter, one possible explanation is that both the development and implementation of the intervention were dominated by a female approach. The intervention was to a large degree developed by women (mainly female researchers and pedagogues involved), it was mainly women who implemented the intervention at school (mostly female teachers) and process evaluation findings indicated that mothers were more involved than fathers at home (more than 80% of the parent answering the process evaluation questionnaire were women). Furthermore, Haug et al. [35] found that boys across Europe and USA were more likely to be overweight than girls, indicating that preventive initiatives may be inadequate and/or less effective for boys. A third explanation may be difference in parental involvement. The process evaluation indicated that parents of girls were more aware of the project compared to parents of boys, which could result in more parental support for the girls. Finally, analyses of the pre-test data from the HEIA study indicate that the girls may have better role models in their mothers compared to boys with regard to weight [29]. Parents, and in particular fathers, should be made aware of their potential to improve as role models [36,37].

When comparing the results from our study with other intervention studies aimed at reducing the consumption of SSB among children/adolescents, only two of the four identified studies reported effects by gender [38-41]. Haerens et al. [40] found no effect, while Singh et al. [41] reported a significant lower intake in the intervention group both for girls and boys. In total, eight [41-48] out of nine [49] identified studies that aimed at reducing the time used for screen activities among both boys and girls aged 9-15 assessed the moderating effect of gender or reported effect in boys and girls separately. Five of the studies [42-44,47,48] reported effects both for boys and girls, while one reported effect for boys only [41]. Harrison et al. [45] found no effect, while Salmon et al. [46] found an effect in the undesired direction in one of the intervention groups. Three of the studies checked the moderating effect of WS for sedentary behaviour [43,45,48]. Harrison et al. [45] found no interaction for screen time. In Planet Health [43], a reduction in TV-viewing predicted obesity change and mediated the intervention effect among the girls. Finally, obese children reported higher screen time at the post-test than overweight and normal weight adolescents in the study by Gentile et al. [48]. These findings are inconsistent and no clear pattern in the behavioural measures emerges, as reported in recent reviews as well [3,9]. The results from our study support that interventions work better for girls than for boys [3,15,16].

Because of the weak evidence of effective school-based obesity prevention interventions, Lytle [4] suggests that it may be time to re-evaluate where the research needs to move. Lytle points out that an investigation of how study participants receive the intervention rarely is examined [4]. The process evaluation in the HEIA study indicates that girls to a larger extent "bring the project home" compared to boys. This result is supported by previous process evaluations [50,51], and studies on gender differences in parent-child communication reporting that girls' self-disclosure at home about every day life is higher than for boys [52]. Parents of boys are more dependent on getting information from others than their sons [53]. This might explain why parents of boys appreciated the fact sheets more than parents of girls. Qualitative studies have found that parents are in need of effective communication strategies about ways to improve positive health behaviours, and that fact sheets may be a useful tool [54,55].

### Strengths and limitations

Our research has some limitations. The SSB consumption variables have not been validated, but our results are in line with data from a national representative study [56]. The measures of sedentary behaviour consisted of single items, resulting in crude estimates only [57]. Still, the mean behavioural outcomes are in line with the trends described by Marshall et al. [58]. The gender differences in time spent on watching TV were small, but boys spent more time on computer/games compared to girls. Furthermore, the test-retest correlation coefficients for the outcome measures were moderate to high (r = 0.46-0.78) [27]. The potential for generalization of our findings is limited because a local sample was recruited from a limited geographic area, mainly in small towns and their close surroundings. The recruitment of schools and participants may have caused a sampling bias, restricting the number of overweight/obese participants and resulting in reduced precision (larger confidence intervals). Finally, some degree of social desirability may be present in the data [59,60]. Still, the effects found should be taken into consideration because of the design of the HEIA study. One of the strengths of the present study is the large sample with objective measures of weight and height. Another strength is that parental education was reported by the parents themselves, and that we were able to collect these data from nearly all the parents giving their adolescent consent to participate in the study, and not only from those parents answering a questionnaire.

## Conclusions

The evaluation of the HEIA study after 8 month revealed that young adolescent boys and girls responded differently to the intervention, and that preventive initiatives thus seem to work better for girls than for boys. Further, it seems important to conduct subgroup analyses to explore potential beneficial or negative effects of interventions. In future research the parental involvement should be evaluated, investigating possible differences in maternal and paternal support and role modelling. More focus on gender and initial WS in the formative evaluation phase of obesity prevention studies also seem warranted in order to enhance the effectiveness of preventive initiatives.

## Competing interests

The authors declare that they have no competing interests.

## Authors' contributions

All authors are responsible for the reported research. M.B. worked on the statistical analyses, wrote the first draft of the manuscript and made the greatest contribution to the paper. All authors participated in designing the study and project planning. N.L. was the project coordinator and participated in all parts of the work. All authors provided critical revision of the paper, and read and approved the final manuscript.
